# Surgical or nonsurgical treatment for nontraumatic rotator cuff tears: Study protocol clinical trial

**DOI:** 10.1097/MD.0000000000020027

**Published:** 2020-05-01

**Authors:** Ding-gen Huang, Ya-li Wu, Peng-fei Chen, Cui-lan Xia, Ze-jin Lin, Jin-qi Song

**Affiliations:** Traumatic Orthopedics, Longhua District Central Hospital, Shenzhen City, Guangdong Province, 518110, China.

**Keywords:** nonsurgery, nontraumatic rotator cuff tear, randomized controlled trial, study protocol, surgery

## Abstract

**Background:**

The optimal treatment for symptomatic, nontraumatic rotator cuff tear is unknown. The primary aim of this randomized controlled trial is to compare functional improvement after surgical and conservative treatment of nontraumatic rotator cuff tears.

**Methods:**

This is a single-centre, randomized clinical trial with a follow-up of 12 months. Patients older than 18 years with magnetic resonance imaging – confirmed nontraumatic rotator cuff tears that are suitable for either surgery or nonsurgery treatment is enrolled. The primary outcome is Constant score. Secondary outcome measures include visual analog scale (VAS) score, patient satisfaction, and American Shoulder and Elbow Surgeons (ASES) score. All scores are assessed by an independent observer who is blinded to the allocation of groups.

**Results:**

The study will provide much needed data on surgical vs nonsurgical treatment for nontraumatic rotator cuff tears. Results of this study may help patients, clinicians, and policy makers assess the pivotal question on comparative effectiveness of surgery vs nonsurgical for rotator cuff tears.

**Trial registration:**

This study protocol was registered in Research Registry (researchregistry5442).

## 1 Introduction

Rotator cuff tear is a common shoulder disorder that lacks a consensus treatment algorithm. It is present in 20% to 54% of persons aged between 60 and 80 years.^[1]^ It has been estimated that rotator cuff tear accounts for 4.5 million patient visits per year and a $3 to $5 billion annual economic burden in the U.S.^[2]^ With increases in the number of physically active aging persons, rotator cuff disease is quickly becoming a major medical and economic burden.

Despite this wide prevalence, controversy exists over the optimal treatment. Nonoperative treatment and surgery are offered to patients with rotator cuff tears with good outcomes for both.^[3]^ However, the evidence base to support surgical versus nonsurgical treatment is quite small and conflicting. Those who favor surgical repair worry that tear progression over time will lead to increased disability with conservative treatment of full-thickness tears.^[4]^ The rotator cuff has limited capabilities for healing without repair, yet conservative treatment often yields an acceptable outcome. Recently, 3 recent prospective randomized controlled trials have compared operative repair to nonoperative treatment in older patients with chronic, degenerative tears of the rotator cuff.^[5–7]^ These studies have generally shown small, nonsignificant differences in favour of surgical repair, and have been unable to provide clear conclusions regarding the preferred treatment. These conflicting results can make decision making regarding the optimal treatment for rotator cuff tears difficult.

The paucity of evidence for operative vs nonoperative treatments for rotator cuff tears is highlighted in the 2012 American Academy of Orthopedic Surgeons Clinical Practice Guidelines, Cochrane reviews, a report by the Agency for Health care Research and Quality, and expert reviews. Thus, a well-conducted randomized clinical trial with an adequate sample size is urgently needed. We undertake a randomized controlled trial to compare outcomes in patients who undertake surgical or nonsurgical treatment for nontraumatic rotator cuff tears. We hypothesize that surgical repair and conservative treatment of nontraumatic rotator cuff tears provide comparable outcomes. Our primary aim is to compare functional outcome after surgical and conservative treatment.

## 2 Materials and methods

This study is performed and reported in accordance with the principles of the Declaration of Helsinki (2000). This study design is a prospective, surgeon- and observer-blinded, randomized controlled trial. Research Ethics Board approval was obtained by the Institutional Review Board in our hospital (JN200213). The study was registered in the Research Registry (researchregistry5442). After written informed consent, all patients are randomized into a surgical and a nonsurgical group.

### 2.1 Patients

**Figure 1:**
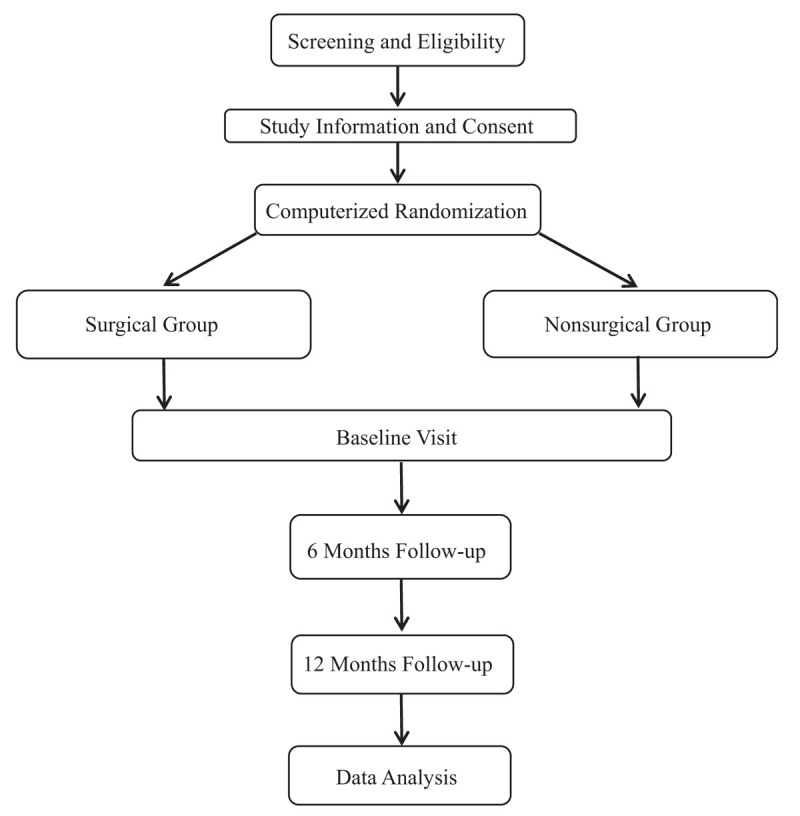
Flow diagram of the study.

Patients with degenerative, nontraumatic full-thickness rotator cuff tears are included in this study from May 2020 to December 2021. Patients should be over 18 years old and can cooperate with us for treatment and postoperative observation. Exclusion criteria are traumatic onset of complaints, previous surgical treatment of the shoulder, frozen shoulder, radiologic and symptomatic osteoarthritis of the glenohumeral or acromioclavicular joint, arthritis/rheumatoid arthritis, diabetes mellitus, cognitive disorders, neurologic disease affecting function of the upper extremity, and language barriers impairing participation (Fig. 1).

### 2.2 Randomization

All patients who met these criteria during the enrollment period are approached about enrollment during a preoperative visit. A computer-generated randomization table is used for patient allocation to 1 of the 2 study groups: the surgical group or the nonsurgical group. Each time a patient is included in the trial, the generated randomized number is assigned accordingly. The randomization assignments are placed in opaque envelopes by research coordinators and then opened after patient enrollment. All staff, including surgeons, nurses and anesthesia staff, are blinded to the contents of the envelope prior to opening, so that patient enrollment cannot be based on group assignment. Demographic information is presented in Table 1.

**Table 1:**
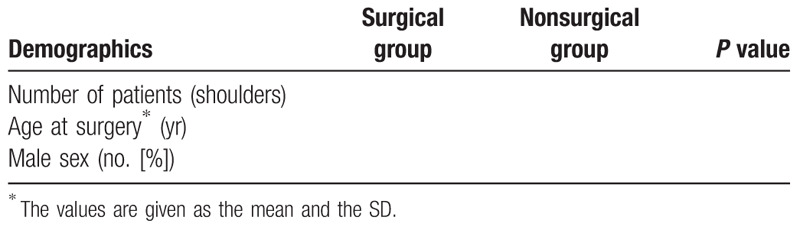
Patient baseline demographics.

### 2.3 Interventions

#### 2.3.1 Surgical group

Procedures in the surgery group are performed by 2 qualified and experienced surgeons. Surgery is scheduled within 6 weeks of inclusion and is done with the patient under general anesthesia, supplement with an interscalene brachial plexus block. The operation is performed in beach chair position using an anterolateral miniopen approach. The coracoacromial ligament is detached from its insertion, and the subacromial bursa is excised. The anteroinferior part of the acromion is removed. The footprint of the rotator cuff on the greater tuberosity is debrided, and a bleeding bony bed is created. Side-to-side repair and repair augmented with bone anchors are performed depending on the shape of the rupture. The deltoid muscle is reattached to the acromion by transosseous refixation.

#### 2.3.2 Nonsurgical group

Treatment in the nonsurgical group consists of subacromial steroid infiltration, physiotherapy, and analgesic medication. After inclusion, patients are given an infiltration in the subacromial space by a posterior approach. If the first infiltration gives no pain relief, a second infiltration will be performed under radiologic or ultrasound guidance. The number of subacromial infiltrations is limited to a maximum of 3. Further conservative treatment options consist of analgesic medication with nonsteroidal anti-inflammatory drugs, paracetamol, or tramadol.

### 2.4 Outcome evaluation

Outcome was assessed in both study groups at 6 and 12 months postoperatively. The Constant score was used as the primary outcome measure. It is a shoulder-specific outcome instrument. Its inter-observer reliability was established in the original paper and a more complete assessment of its reliability in patients with shoulder pathology has been performed by Conboy et al.^[8]^ The system uses a combination of subjective and examiner-derived components to assess shoulder function. A maximal score of 100 points is achievable.

Secondary outcome measures include visual analog scale (VAS) score, patient satisfaction, and American Shoulder and Elbow Surgeons (ASES) score. Pain was evaluated on a VAS with 0 = no pain, and 100 = worst imaginable pain. At the control visits, patients were also asked to grade whether the shoulder was better or worse compared with its preoperative state and if the patients were satisfied or dissatisfied with the treatment outcome. Satisfaction levels are rated using a 100 mm horizontal VAS, for which 0 mm represent completely dissatisfied and 100 mm represent completely satisfied. The self-report section of the ASES score consists of parts for pain and shoulder function, each contributing 50 points to a maximum score of 100. The system's validity, reliability, and responsiveness have been demonstrated.

### 2.5 Statistical analysis

Statistical analyses are conducted using SPSS v22.0 software (IBM, Chicago, IL). All patients in the study are analyzed on the basis of an intention-to-treat principle. This means that for patients in the nonsurgical group who changed treatment, the final score before secondary surgery is carried forward to the 6- and 12-month analyses. Patient characteristics at baseline are compared between groups by *t* tests, chi-squared tests and Mann–Whitney *U* tests. Testing of our null hypothesis (no difference in treatment benefit between groups) is performed by a linear mixed model with an unstructured covariance matrix. The time of follow-up and choice of treatment are used as categorical variables, and an interaction term between time and treatment is included.

### 2.6 Power analysis

With regard to the sample size calculations, the power calculations are based on the assumed changes in the Constant score. On the basis of previous study, the mean score at baseline is assumed to be 50 ± 10 points. At the time of 1-year follow-up, the score is assumed to be 70 points in the best treatment group and 60 points in the worst treatment group. The mean correlation between the measurements during the follow-up is assumed to be 0.40 to 0.50 and the standard deviation of the measurements is assumed to be 20. In an analysis of variance (ANOVA) test with an alpha of 0.05 and a power of 85%, we expect the findings to be significant if the number of subjects per group is 51. Significance is set at *P* < .05. The drop-out rate is assumed to be 15%, and thus the number of subjects per group was 60.

## 3 Discussion

Which patients with rotator cuff tears should have surgical treatment and which should have physiotherapy remains unclear. Early surgical repair is essential for younger, active patients with acute tears, and severe functional deficit.^[9–11]^ In other cases, however, surgery is not indicated and nonoperative treatment may be recommended.^[12]^ This has the advantage of less treatment-related morbidity, but should only be preferred if the short- and long-term results are comparable to those of surgical repair.

Earlier studies regarding either tendon repair or physiotherapy have shown benefit for both approaches.^[13–15]^ However, the studies are difficult to compare because of differing study populations, methods of treatment and evaluation, and periods of follow-up. Most studies of nonoperative treatment are retrospective, present pre-selected groups, and exclude from analysis those patients who were operated upon after failed conservative treatment, the effect of which may have been overestimated.^[16,17]^ This randomized controlled trial is designed to compare outcomes after surgical or nonsurgical treatment of nontraumatic rotator cuff tears.

This trial has some limitations. The results of this study need to be viewed in light of certain limitations. Most importantly, our sample size was small, with 120 patients. Inclusion of patients in this trial may be difficult because most patients have already received conservative treatment or are specifically referred for surgical treatment. Second, the subjects may be exclusively Chinese. Therefore, the data from this clinical trial cannot be applied to other ethnic groups. A third limitation of this study is the follow-up period of only 1 year. Further follow-up is necessary and is underway. However, the study will provide much needed data on surgical vs nonsurgical treatment for nontraumatic rotator cuff tears. Results of this study may help patients, clinicians, and policy makers assess the pivotal question on comparative effectiveness of surgery vs nonsurgical for rotator cuff tears.

## Author contributions

Ding-gen Huang and Ya-li Wu conceived, designed, and planed the study. Ding-gen Huang, Peng-fei Chen, and Ya-li Wu are recruiting the study participants and performing the interventions. Cui-lan Xia and Ze-jin Lin supervised the study. Ding-gen Huang, Ya-li Wu, and Jin-qi Song will interpret and analyze the data. Jin-qi Song drafted the manuscript. Jin-qi Song critically revised the manuscript for important intellectual content. All authors have full access to the manuscript and take responsibility for the study design. All authors have approved the manuscript and agree with submission.
